# Gut Microbiota Dysbiosis Associated With Altered Production of Short Chain Fatty Acids in Children With Neurodevelopmental Disorders

**DOI:** 10.3389/fcimb.2020.00223

**Published:** 2020-05-19

**Authors:** Katarina Bojović, Ður -d ica Ignjatović, Svetlana Soković Bajić, Danijela Vojnović Milutinović, Mirko Tomić, Nataša Golić, Maja Tolinački

**Affiliations:** ^1^Psychiatry Clinic “Dr Selaković”, Belgrade, Serbia; ^2^Department of Biochemistry, Institute for Biological Research “Siniša Stanković”, University of Belgrade, Belgrade, Serbia; ^3^Laboratory for Molecular Microbiology, Institute of Molecular Genetics and Genetic Engineering, University of Belgrade, Belgrade, Serbia

**Keywords:** autism, gut-brain axis, *Lactobacillus*, *Bifidobacterium*, *Clostridium* like species, microbial diversity

## Abstract

While gut microbiota dysbiosis has been linked with autism, its role in the etiology of other neurodevelopmental disorders (NDD) is largely underexplored. To our knowledge this is the first study to evaluate gut microbiota diversity and composition in 36 children from the Republic of Serbia diagnosed with NDD and 28 healthy children. The results revealed an increased incidence of potentially harmful bacteria, closely related to *Clostridium* species, in the NDD patient group compared to the Control group: *Desulfotomaculum guttoideum* (*P* < 0.01), *Intestinibacter bartlettii* (*P* < 0.05), and *Romboutsia ilealis* (*P* < 0.001). On the other hand, significantly lower diversity of common commensal bacteria in the NDD group of patients was noticed. *Enterococcus faecalis* (*P* < 0.05), *Enterococcus gallinarum* (*P* < 0.01), *Streptococcus pasteurianus* (*P* < 0.05), *Lactobacillus rhamnosus* (*P* < 0.01) and *Bifidobacteria* sp. were detected in lower numbers of patients or were even absent in some NDD patients. In addition, butyrate-producing bacteria *Faecalibacterium prausnitzii* (*P* < 0.01), *Butyricicoccus pullicaecorum* (*P* < 0.05), and *Eubacterium rectale* (*P* = 0.07) were less frequent in the NDD patient group. In line with that, the levels of fecal short chain fatty acids (SCFAs) were determined. Although significant differences in SCFA levels were not detected between NDD patients and the Control group, a positive correlation was noted between number of rDNA amplicons obtained with universal primers and level of propionic acid, as well as a trend for levels of total SCFAs and butyric acid in the Control group. This correlation is lost in the NDD patient group, indicating that NDD patients' microbiota differs from the microbiota of healthy children in the presence or number of strong SCFA-producing bacteria. According to a range-weighted richness index it was observed that microbial diversity was significantly lower in the NDD patient group. Our study reveals that the intestinal microbiota from NDD patients differs from the microbiota of healthy children. It is hypothesized that early life microbiome might have an impact on GI disturbances and accompanied behavioral problems frequently observed in patients with a broad spectrum of NDD.

## Introduction

Neurodevelopmental disorders (NDD) [according to Diagnostic and Statistical Manual of Mental Disorders 5th Edition (DSM-5)] (Swedo et al., 2013) or Disorders of psychological development [according to International Classification of Diseases 10th Edition (ICD10)] (WHO, 2015) are a group of disabilities that occur early in childhood, usually at preschool age. Children with NDDs generally have some degree of speech-language pathology, sensory-motor disorders, specific problems in learning and memory and socio-emotional functioning. Autism, which is the most serious neurodevelopmental condition, has attracted the most attention from the scientific community so far. Although the causes of autism are not yet completely understood, it has been suggested that interactions between some genes and environmental factors are needed for full appearance of the disorder (Muhle et al., 2004). The causes of neurodevelopmental disorders other than autism have not been intensively investigated so far.

Lately, co-morbidities, especially gastrointestinal (GI) disturbances, have been recognized as potential risk factors in the development of autism and other NDDs. It has been observed that individuals with autism and other developmental delays are frequently affected by GI disorders like diarrhea, constipation, bloating and gastro-esophageal reflux (Schieve et al., 2012; Chaidez et al., 2014) and that the prevalence of these GI disturbances is higher in children with some developmental disabilities than in children with typical development (Schieve et al., 2012). While it was noticed that GI complications correlate with the severity of behavioral abnormalities, it has been suggested that these co-morbidities could contribute to the manifestation of autism-related behaviors (Horvath and Perman, 2002; Nikolov et al., 2009; Adams et al., 2011; Hsiao, 2014; Tomova et al., 2015).

Many authors have assumed that GI disturbances detected in patients with autism might be linked to an abnormal composition of the gut microbiota and have proposed a connection between the disturbed composition of gut microbiota and autism (Mulle et al., 2013; Borre et al., 2014; De Angelis et al., 2015; Frye et al., 2015; Reddy and Saier, 2015). Several studies have revealed overgrowth of some enteric bacteria, particularly bacteria belonging to *Clostridia* clusters, in children with autism spectrum disorder (ASD) (Finegold et al., 2002; Song Y. L. et al., 2004; Parracho et al., 2005; De Angelis et al., 2013). On the other hand, an imbalance in beneficial bacteria, decrease in *Bifidobacteria* and increase in *Lactobacilli*, was noticed in fecal samples of children with autism (Adams et al., 2011). Although results differed between studies, making it difficult to state what is the “normal” human microbial diversity, the general finding is that microbiota from patients with autism are less diverse and that the balance in beneficial bacteria was disturbed (Krajmalnik-Brown et al., 2015). The significant role of microbiota in CNS functioning and neurodevelopment due to bidirectional communication in the microbiota-gut-brain axis was revealed by Sudo et al. (2004) and Neufeld K. M. et al. (2011). It is postulated that perturbations of initial microbiota colonization can lead to neurochemical disturbances (Borre et al., 2014) through changes in levels of various neuroactive bacterial metabolites, such as serotonin, dopamine, γ-aminobutyric acid, and short chain fatty acids (SCFAs) (Rogers et al., 2016).

Clinical reports have indicated increased incidence of GI disturbances and GI dysbiosis in NDD patients, as well as the improvement of symptoms and the acceleration of recovery after dysbiosis treatment. These observations together with the lack of knowledge about the causes of NDD inspired us to perform the following study. The aim of this study was to evaluate gut microbiota diversity and to identify bacterial strains whose prevalence were statistically different between NDD patients and the Control group, as well as to determine the levels of SCFAs as an indirect measure of balance among the enteric bacteria (Krajmalnik-Brown et al., 2015). The impact of any exclusion diet, such as a gluten-free, casein-free (GFCF) diet, or any medical treatment was excluded since all patients were selected on the first examination. To our knowledge this is the first study of gut microbiota performed on samples from a wide spectrum of NDD patients from Serbia.

## Materials and Methods

### Subjects

Thirty-six pre-adolescent children from the Republic of Serbia diagnosed with NDDs participated in the study. Specifically, 9 patients with expressive and receptive language disorder (ERLD, F80.1 and F80.2), 12 patients with mixed specific developmental disorder (MSDD, F83.0), 10 patients with pervasive developmental disorder non-specified (PDD-NOS, F84.9) and 5 patients with childhood autism (CHA, F84), in total 28 males and eight females between 2 and 9 years of age (median ± SEM, 4.22 ± 0.30 y), participated in the study. The diagnosis was given by a medical doctor specializing in child neuropsychiatry according to the observation of clinical manifestations and anamnesis data given by parents, based on ICD10 criteria, which provides standard criteria for the classification of mental and behavioral disorders. All patients were selected on the first examination, before any medical or nutritional treatment of the developmental disorder was conducted (e.g., didn't follow GFCF diet that could have influence on the gut microbiota composition). From the interview with parents, it was reported that 33% (12/36) of patients suffered from common GI disorders like diarrhea, constipation, gas, bloating, loose stools and undigested food in stool (Supplementary Table 1). Patients hadn't taken probiotics/prebiotics, antibiotics/antimycotics or other supplements for at least 3 months prior to entering the study. All patients were recruited from the Dr. Selaković Psychiatry Clinic, Belgrade, Serbia.

Exclusion criteria for the Control group of 28 unrelated healthy children (19 males and nine females, from 2 to 11 years of age, median ± SEM, 5.96 ± 0.49 y) included the presence of any NDD or GI disorder. Inclusion in the Control group also required that volunteers had not used functional foods (such as probiotics and/or prebiotics or any supplements) or antibiotics/antimycotics for at least 3 months prior to the study.

Written informed consent was obtained from each child's parent prior to inclusion in the study. In addition, a written questionnaire was used during recruitment of volunteers for NDD and Control groups, to assess GI habit and antibiotic usage, and to look for any correlation between the characteristics of individuals and bacterial profiles. This study was carried out under the guidance of the Ethics and Research Committee of the Institute of Molecular Genetics and Genetic Engineering, University of Belgrade (O-EO-002/2015). The study conforms to the World Medical Association's Declaration of Helsinki.

### Collection of Stool Specimens

Three aliquots of stool specimens (5–10 g) were collected in standard sterile sample pots with integral spoon after defecation, immediately placed at −20°C in the participant's freezer and kept there until transported to the laboratory (on ice). The samples were stored at −70°C in the laboratory until the beginning of analysis.

### Denaturing Gradient Gel Electrophoresis (DGGE) Analysis

Extraction of total bacterial DNA from frozen stool samples was done using the ZR Fecal DNA MiniPrepTM kit (Zymo Research, Irvine, CA, USA) according to the instruction manual. DGGE analysis was performed as described previously (Lukic et al., 2013). Fecal samples were studied by performing a PCR-DGGE analysis with extracted genomic DNA as a template (Heilig et al., 2002). Universal primer set F-968-GC and R-1401 targeting the V6-V8 region of 16S rDNA (Nubel et al., 1996), a 16S rDNA-targeted primer set specific for lactobacilli Lab-0159f and Uni-0515-GCr (Heilig et al., 2002) and a primer set specific for *Bifidobacteria* Bif 164-f and Bif 662-CG-r (Satokari et al., 2001) were used to obtain an in-depth view of the microbial diversity in patients' and controls' samples (Table 1). The PCR reaction was performed in a thermal cycler (GeneAmp PCR System 2700, Applied Biosystems, Foster City, CA) programmed as follows: initial denaturation of DNA for 5 min at 95°C, 35 cycles of 30 s at 95°C, 20 s at 56°C, and 40 s at 68°C; and extension of incomplete products for 7 min at 68°C. PCR products were quantified by electrophoresis on a 1% (wt/vol) agarose gel containing ethidium bromide, and visualized by CCD camera Biometra BDR2/5/6 (Bio Doc Analyze). The amplification stage was done using KAPA *Taq* DNA polymerase (KAPA Biosystems, Cape Town, South Africa). PCR amplicons were set on denaturing gradient gel prepared according to Lukic et al. (2013). Optimal separation of the PCR products for the species in the fecal samples was achieved with a 40–80%, 30–60%, and 45–55% urea-formamide denaturant gradient for the Universal primer set, primer set specific for lactobacilli, and primer set specific for *Bifidobacteria*, respectively, increasing in the direction of electrophoresis. A 100% denaturant corresponds to 7 mol/l urea and 40% (vol/vol) formamide. Electrophoresis was performed at a constant voltage of 85 V at temperature of 60°C for 16 h. The amplicons were visualized by AgNO_3_ staining and developing basically as described previously (Sanguinetti et al., 1994).

**Table 1:** Primers used in this study.

**Primer**	**Sequence (5^**′**^ to 3^**′**^)**	**Use**	**Specificity of target**	**References**
F-968-GC	CGC CCG GGG CGC GCC CCG GGC GGG GCG GGG GCA CGG GGG GAA CGC GAA GAA CCT TAC	PCR	V6-V8 region 16S	Nubel et al., 1996
R-1401	CGG TGT GTA CAA GAC CC	PCR	V6-V8 region 16S	Nubel et al., 1996
Lab-0159f	GGA AAC AG (A/G) TGC TAA TAC CG	PCR	Lactobacillus 16S	Heilig et al., 2002
Uni-0515-GCr	ATC GTA TTA CCG CGG CTG CTG GCA CGC CGG GGG CGC GCC CCG GGC GGG GCG GGG GCA CGG GGG G	PCR	Lactobacillus 16S	Heilig et al., 2002
Bif 164-f	GGGTGGTAATGCCGGATG	PCR	Bifidobacterium 16S	Satokari et al., 2001
Bif 662-CG-r	CGCCCGCCGCGCGCGGCGGGCGGGGCGGGGGCACGGGGGGCCACCGTTACACCGGGAA	PCR	Bifidobacterium 16S	Satokari et al., 2001
F-968	AA CGC GAA GAA CCT TA	Sequencing	V6-V8 region 16S	Nubel et al., 1996
Uni-0515r	ATC GTA TTA CCG CGG CTG CTG GCA	Sequencing	Lactobacillus 16S	Heilig et al., 2002
Bif 662-r	CCACCGTTACACCGGGAA	Sequencing	Bifidobacterium 16S	Satokari et al., 2001

### Sequencing of Selected rDNA Amplicons

rDNA amplicons which had incidences that were statistically different between NDD patients and the Control groups (or if a trend existed) were selected for sequencing. The amplicons of interest were excised from the gel and macerated, and the suspension was incubated for 10 min at 98°C (Lukic et al., 2013). After incubation, the suspension was centrifuged and the supernatant (10 μl) was used for PCR amplification with universal primers F-968 and R-1401, *Lactobacillus*-specific primers (LB) Lab-0159f and Uni-0515r and *Bifidobacterium* genus-specific primers (BB) Bif164-f and Bif662-r, using the same PCR program as mentioned above (Table 1). The obtained PCR products were purified using the GeneJET PCR Purification Kit and ligated into the pJET1.2/blunt vector system according to the manufacturer's instructions (Thermo Fisher Scientific, MA, USA). Ligated constructs were transformed in Ca^2+^-induced competent DH5α cells (Hanahan, 1983). Transformants were selected on Luria agar plates containing 100 μg/ml ampicillin. For each excised DNA band two colonies were chosen and plasmids were isolated using the GeneJET Plasmid Miniprep Kit (Thermo Fisher Scientific, MA, USA). The insert-containing pJET1.2/blunt vectors were sequenced with pJET1.2 Forward/Reverse primers (Macrogen Europe Service, Amsterdam, the Netherlands). Sequence annotation and the database searches for sequence similarities were performed using the BLAST tool available online (https://blast.ncbi.nlm.nih.gov/Blast.cgi).

### HPLC–UV Quantitative Determination of Fecal SCFAs

All procedures of SCFA isolation from fecal samples were performed as described previously (De Baere et al., 2013). Quantification of SCFAs in fecal samples was performed by high performance liquid chromatography (HPLC).

Chromatographic separation was tested on a Hypersil Gold aQ column (150 × 4.6 mm i.d.) with a particle size of 3 μm (Thermo Scientific, Voltam, MA, SAD). The HPLC–UV system consisted of an UltiMateTM 3000 LPG 3400 HPLC pump, an UltiMateTM 3000 WPS 3000 TSL autosampler, and an UltiMateTM 3000 DAD 3000 detector, all from Thermo Separations Products (Thermo Scientific, Breda, The Netherlands). The HPLC column was protected by a guard column of the same type. The column was thermostatized at 30°C. The mobile phase consisted of 20 mM of sodium dihydrogen phosphate (NaH_2_PO_4_) (Merck, Darmstadt, Germany) in HPLC water [pH adjusted to 2.2 using phosphoric acid (H_3_PO_4_)] (Merck) (A) and HPLC grade acetonitrile (C_2_H_3_N) (Sigma-Aldrich, St. Louis, Missouri, USA) (B). A gradient elution was performed as shown in Supplementary Table 2. The UV detector was set at a wavelength of 210 nm. Data processing was performed using Chromeleon version 6.8 software (Thermo Fisher Scientific, MA, USA).

For quantification of SCFAs the external calibration standards curve method was used. Calibration standards were prepared with concentrations ranging from 0.5 mM to 50 mM for acetic acid (AA), propionic acid (PA), butyric acid (BA), and succinic acid (SA) as internal standards.

Each analysis day, standards for calibration curves were freshly prepared in HPLC grade water. Samples were spiked at an individual organic acid concentration of 25 mM. All measurements were run in triplicate. Standards: acetic, propionic, butyric and succinic acid were purchased from Sigma–Aldrich, USA.

Fecal concentrations of SCFA were calculated by using the following equation: SCFA (AA, PA, BA) = (organic acid in fecal sample × 6 × 10^−3^)/(succinic acid in fecal sample × mass of fecal sample) × 1000 [mmol/kg].

### Statistical Analyses

Data are presented as mean ± SEM for continuous variables and as percentages for categorical variables. Results were considered statistically significant at *P* < 0.05, P value between 0.05 and 0.1 was considered a trend toward significant difference. Statistical analyses were performed using GraphPad Prism v5 software (San Diego, California, USA).

Comparisons between the two groups (NDD patient *vs*. Control group) were performed with a Student's *t*-test and Pearson's Chi-square (with Yates‘ correction for continuity) test for quantitative and categorical variables, respectively. One-way ANOVA followed by a Tukey *post-hoc* test were used for the analyses of differences between groups with a particular NDD diagnosis and the Control group.

Similarities between samples (NDD patient vs. Control group) were determined by calculating similarity indices based on the Dice similarity coefficient. Two identical profiles create a value of 100%, whereas two profiles without common bands result in a value of 0%. Dice similarity coefficients were used to compare DGGE band profiles between individuals within the same group and between members of the NDD patient and Control group, for every possible pair of samples. The Dice similarity coefficient was calculated using the formula: D_sc_=[2j/(a+b)]^*^100 (%), where “a” is defined as the number of bits set to “1” in sample A, “b” as the numbers of bits set to “1” in sample B and “j” as the numbers of bits that are “1” in both samples A and B. D_sc_=100% presents identical DGGE profiles, while D_sc_=0% refers to completely different DGGE profiles.

## Results

### Gender Bias and Increased Incidence of GI Disturbances in NDD Patients

In our study a gender bias was noticed, with the gender ratio being 3.5:1 (28 males: 8 females) in the patient group and 2.1:1 (19 males: 9 females) in the selected corresponding Control group. According to the parents' reports, 33% (12/36) of NDD patients in this study frequently had one or more GI disorders like diarrhea, constipation, gas, bloating, loose stools and undigested food in the stool. The relevant symptoms are given in Supplementary Table 1.

### Dice Similarity Coefficient Analysis of Gut Bacteria in NDD Patients and Healthy Children

**Figure 1:**
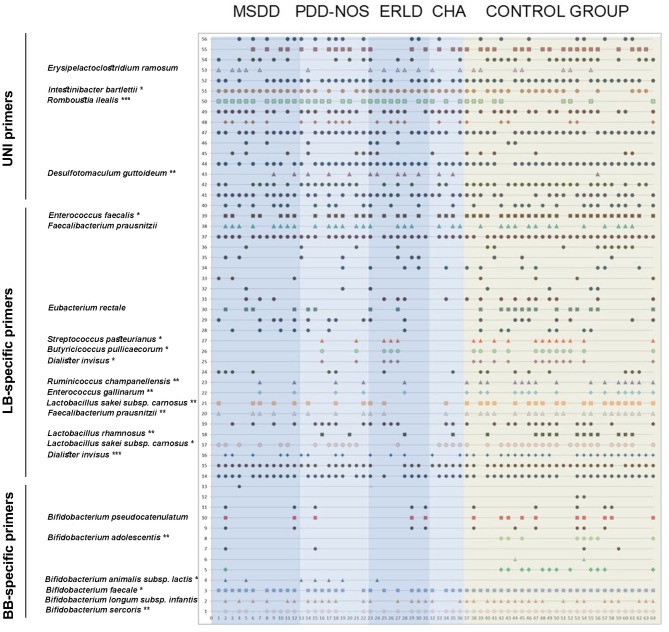
The identity of the rDNA clones obtained from DGGE rDNA amplicons related to the individual children. The numbers on the y-axis correlate to the numbers of the rDNA amplicons observed. The rDNA amplicons whose incidences were statistically different between NDD patient and Control groups (or if trend existed) were excised, cloned, and sequenced and identities of those bacterial species are displayed. The numbers on the x-axis correspond to the numbers of lanes. Each lane represents sample of an individual child. Total of 64 subjects (36 NDD patients and 28 healthy controls) was analyzed. Each symbol represents a corresponding band on DGGE gel. MSDD, mixed specific developmental disorder; PDD-NOS, pervasive developmental disorder-non-specified; ERLD, expressive and receptive language disorder; CHA, childhood autism; NDD, neurodevelopmental disorder.

In order to compare the fecal microbiota of children with NDD and healthy children, DGGE analyses of rDNA amplicons obtained with three sets of primers (universal, *Lactobacillus* (LB) and *Bifidobacteria* (BB) specific primers) were performed (Figure 1).

**Figure 2:**
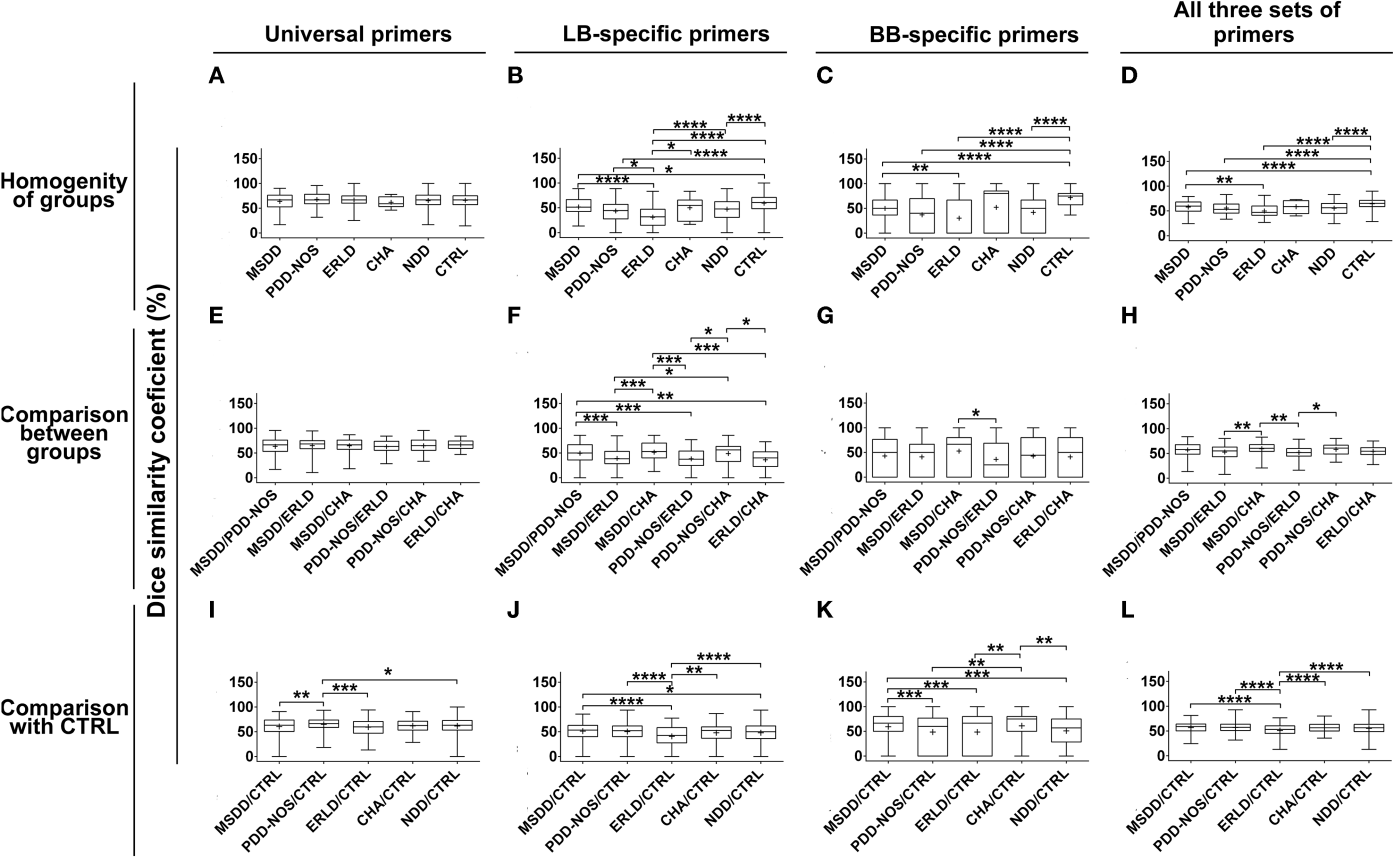
Box-plot diagram based on Dice similarity coefficient. Homogeneities of the groups and similarities between the groups were evaluated by Dice similarity coefficient (%). Homogeneities of the groups were evaluated by comparing DGGE profiles obtained by universal, *Lactobacillus* (LB)-, *Bifidobacteria* (BB)-specific primers and all three sets of primers together **(A–D)** within Control (CTRL) and particular NDD groups. Similarities between particular NDD groups and similarities of NDD groups with the CTRL group were evaluated by comparing DGGE profiles of samples from particular NDD groups, obtained by universal, LB-, BB-specific primers and all three sets of primers together, between NDD groups **(E–H)** and with DGGE profiles of the CTRL group **(I–L)**, respectively. Relevant statistical values of Dice analysis are presented in Supplementary Table 3. *p < 0.05; **p < 0.01; ***p < 0.001; ****p < 0.0001.

DGGE profiles obtained with universal primers showed large interpersonal diversity in all groups. Therefore, Dice similarity coefficients didn't show statistically significant differences concerning homogeneity of the groups or similarity between groups (Figures 2A,E). When LB-specific and BB-specific primer sets were used a high similarity of DGGE profiles was observed within the Control (CTRL) group (59.30 ± 15.21 and 71.88 ± 16.16, respectively), while the lowest similarity was scored within the ERLD group (31.12 ± 22.3 and 30.13 ± 37.50, respectively) (Figures 2B,C). When amplicons obtained with LB-specific primers were analyzed, the results revealed that among the NDD patients' groups the most homogenous groups were the MSDD (51.77 ± 18.74) and CHA groups (50.17 ± 23.56) (Figure 2B). With amplicons obtained using BB-specific primers, the most homogeneous group among NDD patients was CHA (52.00 ± 45.41) (Figure 2C). When the amplicons obtained using all three sets of primers were analyzed, a high similarity of DGGE profiles was observed within the CTRL group (64.57 ± 9.35), and the lowest similarity was observed within the ERLD group (49.90 ± 13.64). A lower level of homogeneity than CTRL, but similar to each other, was noticed among other patient groups (MSDD, 57.80 ± 12.54; PDD-NOS, 55.65 ± 12.47, and CHA 58.53 ± 12.59) (Figure 2D).

A Dice similarity coefficient, using LB-specific primers and BB-specific primers, revealed that the DGGE profiles of MSDD and CHA groups were similar (51.55 ± 19.89 and 52.85 ± 11.55, respectively) (Figures 2F,G). Analysis with all three sets of primers revealed that MSDD and CHA were similar (59.58 ± 12.16), as well as PDD-NOS and CHA (58.29 ± 11.55), and MSDD and PDD-NOS (56.84 ± 14.17), while the similarities of MSDD, PDD-NOS and CHA with ERLD were lower (52.74 ± 14.50, 51.90 ± 11.20, and 54.15 ± 10.58, respectively) (Figure 2H).

According to analyses of DGGE profiles obtained with universal, LB-specific, and BB-specific primers, so all three sets of primers together, the Dice similarity coefficient revealed that the ERLD group was least similar to the CTRL (59.29 ± 15.76; 40.64 ± 20.99; 46.16 ± 35.38; 50.88 ± 14.32, respectively) (Figures 2I–L). When universal primers were used, the Dice similarity coefficient showed that the PDD-NOS group was the most similar to the CTRL (64.89 ± 13.03) (Figure 2I), LB-specific primers showed that the MSDD group is the most similar to the CTRL (51.18 ± 15.97) (Figure 2J), while DGGE profiles obtained with BB-specific primers revealed that the CHA group is the most similar to the CTRL (57.35 ± 32.66) (Figure 2K). Finally, when all three sets of primers were used, Dice similarity coefficients revealed that MSDD (56.95 ± 10.59) and PDD-NOS (57.00 ± 10.75) are the most similar to the CTRL (Figure 2L). Values of dice analyses are presented in Supplementary Table 3.

### Microbial Diversity Comparisons

Microbial diversity was evaluated according to a range-weighted richness index (Rr) which represents the total number of rDNA amplicons obtained with PCR-DGGE analysis, multiplied by the percentage of denaturing gradient, in order to describe the total diversity of the sample analyzed, according to the following formula:

$$\begin{array}{l} \text{Rr }=\;{\text{N}}^{2}\text{x Dg} \end{array}$$

where N represents the total number of rDNA amplicons in the pattern, and Dg the denaturing gradient comprised between the first and the last band of the pattern. A higher Rr value represents higher diversity. Comparisons between Control and NDD patient groups were performed.

**Figure 3:**
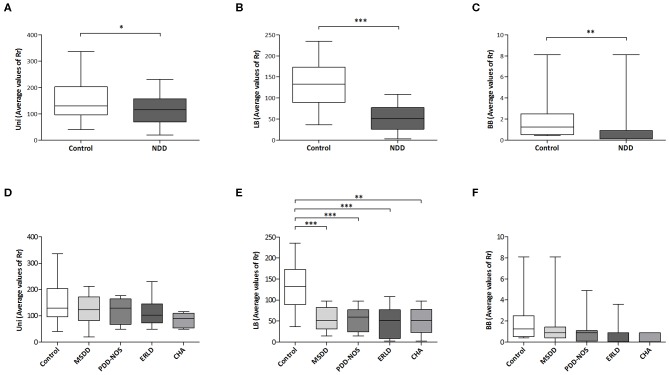
Microbial diversity comparison. Student's unpaired *t*-test was used to compare mean values of range-weighted richness index (Rr) derived from DGGE patterns obtained with universal (Uni) primers **(A)** and *Lactobacillus* (LB)-specific primers **(B)** between NDD patient and Control group. Due to absence of normal distribution of range-weighted richness indexes derived from DGGE patterns obtained with *Bifidobacteria* (BB)-specific primers, difference between groups was assessed by non-parametric Mann–Whitney *U* test **(C)**. Comparison of mean values of Rr derived from DGGE patterns obtained with universal **(D)** and LB-specific primers **(E)** between particular NDD diagnoses and Control group was performed with one-way ANOVA followed by Tukey *post-hoc* test, while for comparison of Rr derived from DGGE patterns obtained with BB-specific primers Kruskal-Wallis test was used **(F)**. MSDD- mixed specific developmental disorder, PDD-NOS- pervasive developmental disorder-non-specified, ERLD, expressive and receptive language disorder; CHA, childhood autism; NDD, neurodevelopmental disorder. *p < 0.05; **p < 0.01; ***p < 0.001.

It was observed that in the analyzed groups a significant difference in Rr exists between DGGE patterns obtained with universal primers (*P* < 0.05) (Figure 3A), LB-specific primers (*P* < 0.001; Figure 3B) and BB*-*specific primers (*P* < 0.01; Figure 3C).

One-way ANOVA revealed statistically significant between-group differences in average Rr values for DGGE patterns obtained with LB-specific primers (*P* < 0.001). In addition, *post hoc* analyses of Rr values derived from DGGE patterns obtained with LB-specific primers revealed significant differences in Rr between the Control (131.89 ± 10.66) and each of the NDD patient groups: MSDD (54.60 ± 8.26, *P* < 0.001), PDD-NOS group (52.98 ± 8.92, *P* < 0.001), ERLD (45.93 ± 12.25, *P* < 0.001), and CHA (50.52 ± 15.15, *P* < 0.01; Figure 3E), indicating significantly lower diversity in NDD patient groups compared to the Control group. Significant differences in Rr derived from DGGE patterns obtained with universal primers between NDD patient groups and the Control group were not detected, while for Rr derived from DGGE patterns obtained with BB-specific primers a trend was noted (*P* = 0.08) (Figures 3D,F, respectively).

### Identification of Key Bacterial Species Linked to NDD

rDNA amplicons which had incidences that were statistically different between NDD patients and the Control group (or if trends existed) were selected for sequencing. The results of sequencing and P values are presented in Table 2. The results revealed lower diversity of regular constituents of gut microbiota and higher incidence of several potentially harmful bacteria in the gut microbiota of NDD patients.

**Table 2:** Identification of bacterial species according to sequencing of selected rDNA amplicons.

**No rDNA amplicon**	**Bacterial species**	***P***
**BACTERIAL SPECIES OBTAINED WITH UNIVERSAL PRIMERS**		
53	*Erysipelatoclostridium ramosum* strain JCM 1298	0.34
51	*Intestinibacter bartlettii* strain WAL 16138	<0.05
50	*Romboutsia ilealis* strain CRIB (2)	<0.001
43	*Desulfotomaculum guttoideum* strain DSM 402	<0.01
**BACTERIAL SPECIES OBTAINED WITH LB-SPECIFIC PRIMERS**		
39	*Enterococcus faecalis* strain NBRC 100480	<0.05
30	*Eubacterium rectale* strain ATCC 33656	0.07
27	*Streptococcus pasteurianus*	<0.05
26	*Butyricicoccus pullicaecorum* strain 25-3	<0.05
25	*Dialister invisus* strain JCM 17566 (1)	<0.05
23	*Ruminococcus champanellensis* strain 18P13	<0.01
22	*Enterococcus gallinarum* strain LMG 13129	<0.01
21	*Lactobacillus sakei* subsp. *carnosus* strain CCUG 31331	<0.01
38	*Faecalibacterium prausnitzii* strain ATCC 27768	<0.01
18	*Lactobacillus rhamnosus* strain NBRC 3425	<0.01
17	*Lactobacillus sakei* subsp. *carnosus* strain CCUG 31331 (2)	<0.05
16	*Dialister invisus strain* JCM 17566 (2)	<0.001
**BACTERIAL SPECIES OBTAINED WITH BB-SPECIFIC PRIMERS**		
10	*Bifidobacterium pseudocatenulatum* strain B1279	0.08
8	*Bifidobacterium adolescentis* strain ATCC 15703	<0.01
4	*Bifidobacterium animalis* subsp. *lactis* strain YIT 4121	<0.05
3	*Bifidobacterium faecale*	<0.05
2	*Bifidobacterium longum* subsp. *infantis* strain ATCC 15697	0.21
1	*Bifidobacterium stercoris* strain Eg1 16S ribosomal	<0.01

*DGGE analysis of rDNA amplicons obtained using DNA isolated from fecal samples of 36 children with NDD and 28 healthy controls, as templates and three sets of primers (universal, LB and BB-specific primers) complementary to 16S rDNA regions was performed. rDNA amplicons whose incidences were statistically different (or if trend existed) between NDD patient and Control group were selected for sequencing. Numbers correspond to the rDNA amplicons on the Figure 1. Comparisons between NDD patient and Control group were performed with Pearson's Chi-square test. Results were considered statistically significant at P < 0.05*.

### Higher Incidence of Potentially Harmful Bacteria in Fecal Microbiota of NDD Patients

Several bacterial species were found more frequently in the patients' groups compared to the control samples, specifically *Desulfotomaculum guttoideum* (*P* < 0.01), *Intestinibacter bartlettii* (previously known as *Clostridium bartlettii*) (*P* < 0.05) and *Romboutsia ilealis* (*P* < 0.001; Table 2). *D. guttoideum* was present in the highest percentage in ERLD samples (55.56%, *P* < 0.01), in 40% (*P* = 0.08) of CHA and in 30% (*P* = 0.08) of the PDD-NOS patients, in comparison to very low occurrence in the control samples (3.6%) (Supplementary Table 4). *Intestinibacter bartlettii* was present in all (100%) of the MSDD (*P* < 0.05), ERLD (*P* = 0.09) and CHA (ns) patients' samples, while it was noticed in 64.3% of the control samples. *Romboutsia ilealis* was present in 91.7% (*P* < 0.01) of MSDD, 88.9% (*P* = 0.01) of ERLD, 80% (*P* < 0.05) of the PDD-NOS and 60% (ns) of CHA patients, but in only 32% of the control samples. In addition, a high incidence of *Erysipelatoclostridium ramosum* (previously known as *Clostridium ramosum*) was noted in the ERLD (55.6%, ns), MSDD (41.7%, ns), and CHA (40%, ns) patients' groups compared to 25% in the control samples.

It was noted that *I. bartlettii* (100%, *P* < 0.05) and *R. ilealis* (91.7%, *P* < 0.01) were present in a higher number of MSDD patients compared to the Control group, while a high incidence of *R. ilealis* (80%, *P* < 0.05) and *D. guttoideum* (30%, *P* = 0.08) was observed in the PDD-NOS group. High incidences of all identified *Clostridium*-related species [*D. guttoideum* (55.6%, *P* < 0.01), *R. ilealis* (88.9%, *P* < 0.05), *I. bartlettii* (100%, *P* = 0.09) and *E. ramosum* (55.6%, ns)] were detected in the ERLD group of patients. Although in the CHA group a significant difference in comparison to the Control group wasn't achieved, probably due to the small number of samples in this group, high incidences of *D. guttoideum* (40%, *P* = 0.08), *R. ilealis* (60%, ns), *I. bartlettii* (100%, ns), and *E. ramosum* (40%, ns) were also noticed.

### Lower Incidence of Lactic Acid Bacteria (LAB), Bifidobacteria, and Butyrate-Producing Bacteria in Fecal Microbiota of NDD Patients

*Enterococcus faecalis* was present in 89.3% of the control samples, while it was found in only 58.3% (*P* < 0.05) of NDD patients' samples, particularly in 58.3% (*P* = 0.07) of MSDD and 55.6% (*P* = 0.08) of ERLD patients' samples. *Enterococcus gallinarum* wasn't detected in CHA patients' samples (*P* = 0.08) and was present in only 11.1% of the ERLD patients (*P* = 0.06) and 16.7% of the MSDD samples (*P* = 0.07), compared to 53.6% in the control samples. Moreover, *Streptococcus pasteurianus* was less present in the patients' groups (*P* < 0.05), being completely absent in the MSDD and CHA group (*P* < 0.05 and ns, respectively). Among the *Lactobacillus* genus, *Lactobacillus rhamnosus* (*P* < 0.01) and *L. sakei* (*P* < 0.01) were found to be less frequently present in the NDD patient group. *L. rhamnosus* was completely absent in MSDD (*P* < 0.01) and present in a low percent of ERLD (11.1%, ns), CHA and PDD-NOS (20%, ns) patients, while it was present in 42.9% of the control samples.

A complete absence of *Bifidobacterium* sp. was noticed in 12% of NDD patients, while such absolute deficit wasn't observed in any of the control samples. Particularly, *B. faecale* (*P* < 0.05) was found in all of the control samples while it was absent in 22.2% of patients' samples. *B. adolescentis* was present in 28.6% of children in the Control group (*P* < 0.01), but was not detected in any of the patients' samples. *B. stercoris* (*P* < 0.01) and *B. pseudocatenulatum* (*P* = 0.08) were also less frequently present in the NDD patient group. *B. pseudocatenulatum* was absent in all of the CHA patients and was present in only 10% of PDD-NOS patients, while among the Control group it was present in 35.7% of samples. Although not significant, a lower incidence of *B. longum* subsp. *infantis* was observed in the patients' groups. In contrast, *B. animalis* subsp. *lactis* was observed to be present in all patients' groups except CHA and it was present in the highest percentage in PDD-NOS samples (40%, *P* < 0.01), while not detected in any of the control samples (Supplementary Table 4).

Similarly, the butyrate producing bacteria *Faecalibacterium prausnitzii* (*P* < 0.01), *Butyricicoccus pullicaecorum* (*P* < 0.05) and *Eubacterium rectale* (*P* = 0.07) were less frequent in the patients' groups (Table 2). *Faecalibacterium prausnitzii* was present in only 20% (ns) of CHA and 22.2% (*P* < 0.05) of ERLD patients' samples while it was present in 67.9% of the control samples. *Butyricicoccus pullicaecorum* was completely absent in CHA (ns) and MSDD patients' samples (*P* < 0.05), while it was present in 42.9% of the control samples. *Eubacterium rectale* was not detected in any of the CHA patient's samples (ns) and was detected in only 20% (ns) of PDD-NOS samples, while it was present in 46.4% of the control samples.

In addition, *Ruminococcus champanellensis* (*P* < 0.01) and *Dialister invisus* (*P* = 0.001) were also less frequent in the patient group. *R. champanellensis* was absent in CHA (*P* = 0.08) and detected in only 11.1% (*P* = 0.06) of ERLD samples, while it was present in 53.6% of the control samples. *D. invisus* was present in 41.7% (*P* < 0.05) of the MSDD samples, 40% of the PDD-NOS and CHA samples (*P* < 0.05 and ns, respectively) and 33.3% (*P* < 0.05) of ERLD samples, while it was present in 82.1% of the control samples (Supplementary Table 4).

Although significant differences between CHA and the Control group weren't detected, deficiency of all of the identified constituents of normal gut microbiota was observed in this group, the most prominent being complete absence of: *E. rectale, S. pasteurianus, B. pullicaecorum, R. champanellensis, E. gallinarum, B. pseudocatenulatum* and *B. adolescentis*.

Furthermore, the complete absence of *S. pasteurianus, B. pullicaecorum, L. rhamnosus and B. adolescentis* was noted in MSDD patients.

### Report of Prominent Case

The most striking example of microbiota diversity disturbance associated with NDD in our study was the case of a 2-year-old boy, diagnosed as MSDD (Figure 1: patient number 7) whose DGGE pattern obtained with universal primers showed extremely decreased diversity compared to samples of other patients and controls. The two strains that were most present in this patient's sample were the *Clostridium*-like strains *I. bartlettii* and *E. ramosum*. PCR-DGGE analyses have shown a complete absence of *Bifidobacterium* sp., *L. rhamnosus, E. rectale, B. pullicaecorum*, and *D. invisus* in this individual's sample. The patient's parents reported periodic constipation, while developmental delay, hypotony, and anxiety were clinically observed.

### Analysis of SCFAs in Fecal Samples of NDD Patients and Healthy Children

**Figure 4:**
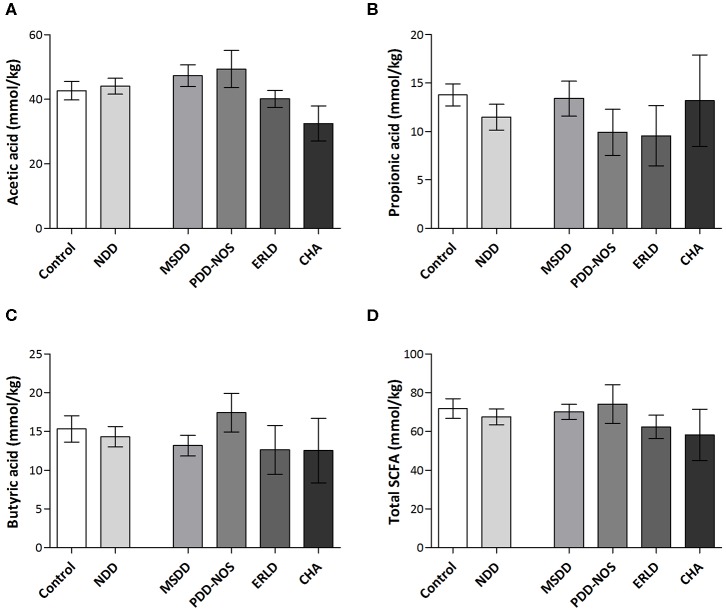
Amounts of SCFAs in fecal samples of NDD patients and healthy children. The amounts of acetic acid **(A)**, propionic acid **(B)**, butyric acid **(C)**, and total SCFAs **(D)** in NDD, Control group and particular NDD diagnoses groups were presented in mmol/kg of feces. Quantification of SCFAs in fecal samples was performed by HPLC analysis. Results are presented as Mean ± SEM. MSDD, mixed specific developmental disorder; PDD-NOS, pervasive developmental disorder-non-specified; ERLD, expressive and receptive language disorder; CHA, childhood autism; NDD, neurodevelopmental disorder.

The amount of SCFAs in fecal samples, including acetate, propionate and butyrate, were analyzed. The results revealed that the SCFA content largely varied within groups. Interestingly, the values of total SCFAs, as well as levels of acetic and butyric acid, were the lowest in the CHA patient group, although the differences between patient and control groups were not significant, presumably due to the small number of CHA patients (Figure 4).

**Figure 5:**
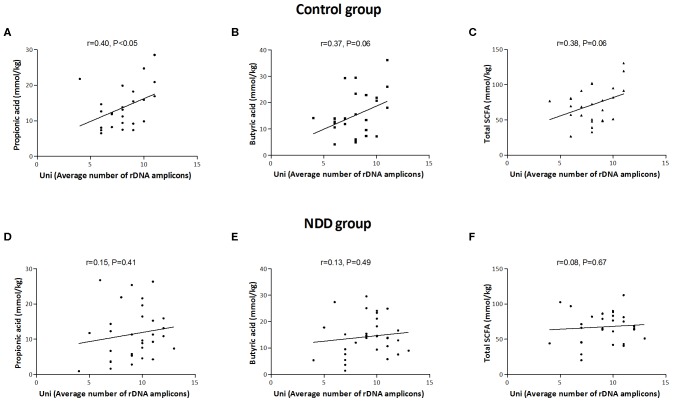
Correlation between average number of rDNA amplicons and amounts of SCFAs in Control and NDD patient group. Average number of rDNA amplicons obtained with universal primers was correlated with amount of propionic acid **(A)**, butyric acid **(B)**, and total SCFAs **(C)** in Control group and in NDD group **(D–F)**. Each dot corresponds to one subject. The line denotes linear regression of individual data point; r stands for Pearson's correlation coefficient. *P* < 0.05 was considered statistically significant; NDD, neurodevelopmental disorder.

Correlation analysis revealed that a significant positive correlation exists between number of rDNA amplicons obtained with universal primers and the level of propionic acid (*r* = 0.40, *P* < 0.05), as well as a trend for level of total SCFAs (*r* = 0.38, *P* = 0.06) and butyric acid (*r* = 0.37, *P* = 0.06) in the Control group. This correlation is lost in the NDD patient groups, indicating that the microbiota of NDD patients differs from microbiota of healthy children in the presence and/or number of strong SCFA-producing bacteria (Figure 5).

## Discussion

Recently, differences in composition of gut microbiota from children with autism and healthy children were intensively studied (Hughes et al., 2018). However, studies evaluating diversity and composition of gut microbiota in other NDDs are lacking. Hence, according to our knowledge, this is the first study to evaluate the specificities of gut microbiota in a broad spectrum of NDD patients. The impact of any exclusion diet, such as a GFCF diet or of any medical treatment was excluded here because all patients were selected on the first examination.

A gender bias toward boys was noticed in the patients' groups (7:2) in accordance with observed diagnosis sex bias in NDDs such as autism (4:1) (Scott et al., 2002) and intellectual disability/developmental delay 2:1 (Gillberg et al., 2006; Ropers, 2008; Polyak et al., 2015). As reported by parents, 1/3 (33%) of NDD patients in this study frequently had one or more GI disorders including diarrhea, constipation, gas, bloating, loose stools and undigested food in stools, in accordance with clinical medical records describing the occurrence of GI symptoms in 24% of ASD patients (Molloy and Manning-Courtney, 2003) and in 22% of PDD patients (Nikolov et al., 2009).

According to the Dice similarity coefficient acquired when DGGE profiles obtained with LB- and BB- specific primers were compared, as well as when DGGE profiles obtained with all three sets of primers were compared together, the microbiota of the Control and CHA groups were the most homogenous, while microbiota of the ERLD group was the most heterogeneous one, indicating large interpersonal diversity in this patient group. DGGE profiles of the MSDD group also showed high homogeneity when LB-specific primers were used.

When DGGE profiles obtained with all three sets of primers were compared between groups, a high similarity was observed between CHA and PDD-NOS (also known as “atypical autism”) (Volkmar and Reichow, 2013) groups, which are also the most similar groups of NDD according to clinical/diagnostic assessments. High similarity was noticed between MSDD and PDD-NOS, and MSDD and CHA groups. The similarities of all these three groups (CHA, PDD-NOS, and MSDD) with the ERLD group was low. This is in concordance to the clinical similarity of MDD, CHA and PDD-NOS, which are more complex and severe disorders than ERLD, having similar levels of functionality and more complex and severe autism-like symptoms compared to ERLD (WHO, 2015). Similarity of microbiota composition in patient groups which have similar neurological and behavioral symptoms might lead to the assumption that intestinal microbiota have a significant role in the pathophysiology of NDDs.

The microbiota of ERLD patients showed the smallest degree of similarity with the microbiota of the Control group. This result indicates that modification of intestinal microbiota, in children with less serious NDD, such as ERLD, might be important. This assumption is in agreement with the observed efficacy of rehabilitation and decline of autism-like symptoms in this group of patients after medical treatment of dysbiosis in clinical practice.

Analysis of DGGE profiles obtained with universal primers revealed that the microbiota of PDD-NOS patients is the most similar to the microbiota of the Control group, which might indicate that disturbances in composition of these bacteria might have lower significance in this group of patients. However, *R. ilealis* (80%, *P* < 0.05) was significantly more present in this group of patients compared to the Control group. Further, DGGE profiles obtained with LB-specific primers showed that the MSDD group is the most similar to the Control group, although complete absence of *S. pasteurianus, B. pullicaecorum, L. rhamnosus* and lower incidence of *D. invisus* was noted in this group. Finally, even though analysis of the DGGE profile obtained with BB-specific primers showed that the CHA group is the most similar to the Control group, the lowest number of *Bifidobacteria* and complete absence of *B. pseudocatenulatum* and *B. adolescentis* were noted in this group. We assume that this similarity is obtained according to the absence of *B. animalis*, which is also absent in the Control group, while present in all other NDD groups.

The results of this study reveal that diversity of common commensal bacteria in the patients' groups was significantly lower compared to the Control group, while the incidence of several potentially harmful bacteria was higher. Microbial species such as *Desulfotomaculum guttoideum, Intestinibacter bartlettii* and *Romboutsia ilealis*, all closely related to *Clostridium* clusters (Stackebrandt et al., 1997; Gerritsen et al., 2014), were found to be more frequently present in the NDD patients group. *D. guttoideum* (reclassification to the *Clostridium* genus proposed two decades ago based on 16S rDNA analysis) (Stackebrandt et al., 1997; Castro et al., 2000) is a sulfate-reducing bacterium similar to *Desulfovibrio*, which has been found in increased incidence in children with autism (Finegold et al., 2010, 2012; Tomova et al., 2015). It was suggested that sulfate-reducing organisms could account for much of the abnormality in sulfur metabolism seen in autistic subjects, such as low blood levels of sulfur and high urinary excretion, reduced methylation capacity, decrease in trans-sulfuration and chronic oxidative stress (Finegold et al., 2012). According to increased *Desulfovibrio* and *Clostridia* species in the microbiota of children with autism it was speculated that these bacteria could be involved in development of autism and that these bacteria could contribute in some way to autism pathogenesis (Tomova et al., 2015).

Several research groups that have analyzed microbiota in children with autism have detected overgrowth of *Clostridium* spp. (Finegold et al., 2002; Song Y. et al., 2004; Parracho et al., 2005; De Angelis et al., 2013). It was hypothesized that regressive autism could be a consequence of gut microbiota dysbiosis caused by antimicrobial therapy. Overgrowth of antimicrobial resistant microorganisms, such as *Clostridium tetani*, leads to elevated production of the neurotoxin *p*-cresol or toxic metabolites (phenols and indole derivatives) which cause anxiety-like behavior (Bolte, 1998; Finegold et al., 2012; Hsiao et al., 2013). In line with that, it was reported that cognitive and social functions in ASD patients were improved to some extent after vancomycin treatment (Sandler et al., 2000).

In addition, it was revealed that *I. bartlettii*, previously known as *Clostridium bartlettii* isolated from the feces of two autistic male children (Song Y. L. et al., 2004), is a strong producer of trans-3-indolylacrylic acid (IAA) (Russell et al., 2013), which can be transformed by glycine conjugation (Clayton, 2012) to Indolyl-3-acryloylglycine (IAG), a putative ASD diagnostic urinary marker (Shattock and Whiteley, 2002; Bull et al., 2003). Moreover, *Erysipelatoclostridium ramosum* (previously known as *Clostridium ramosum*), which was noted more frequently in the ERLD, MSDD, and CHA patients compared to the Control group in this study, was formerly isolated from the stool specimens of children with autism (Finegold et al., 2002).

Even though the interpersonal diversity of both Control and NDD patient groups was large, a significant decrease in diversity of LAB was detected. The identified strains that were present in control samples with greater incidence compared to patients' samples were common commensal bacteria and butyrate producing bacteria. *En. faecalis, En. gallinarum*, and *S. pasteurianus*, which are part of the commensal microbiota (Layton et al., 2010; Alex et al., 2013; Rajilic-Stojanovic and De Vos, 2014), were detected in low numbers or even absent in some NDD patients' groups. This is in accordance with literature data reporting decreased levels of LAB (*Enterococcus* sp., *Lactobacillus* sp., *Streptococcus* sp., and *Lactococcus* sp.) in the fecal samples of children with autism and PDD-NOS (De Angelis et al., 2013). Among *Lactobacillus* sp., *Lactobacillus rhamnosus* was found to be less frequently present in the NDD patient group. It was shown that ingestion of *L. rhamnosus* JB-1 has an impact on the regulation of emotional behavior associated with anxiety and depression in normal, healthy mice, through modulation of central GABA receptor expression (Bravo et al., 2011).

Similarly, decreased frequency of *Dialister invisus* was noticed in the NDD patient group. Although *D. invisus* species are typically isolated from the oral cavity, they have also been detected in samples of the normal gastrointestinal microbiota (Rajilic-Stojanovic et al., 2007). Decreased populations of *D. invisus* have been reported in autistic children and its importance in contributing to protective flora was suggested (Finegold et al., 2010).

Moreover, a significant decrease of *Bifidobacteria* was noticed, including the complete absence of any detectable *Bifidobacteria* in 12% of NDD patients, while such an absolute deficit wasn't observed in any of the control samples. This is in accordance with previous reports where lower levels of *Bifidobacterium* spp. were found in samples from children with autism (Adams et al., 2011; Wang et al., 2011; De Angelis et al., 2013). It is important to emphasize that *Bifidobacteria* are among the first microbial colonizers of the gut immediately after birth (Turroni et al., 2012), a dominant bacterial genus in the infant gut microbiota (Harmsen et al., 2000; Matamoros et al., 2013), and significant member of commensal gut bacteria in adults (Ringel-Kulka et al., 2013; Chaplin et al., 2015). Their beneficial role for the host is assigned to digestion of indigestible polysaccharides, production of B-group vitamins, competitive pathogen exclusion, production of antimicrobial agents, and suppression of inflammatory responses (Deguchi et al., 1985; Sonnenburg et al., 2006; Lee and O'sullivan, 2010; Miyauchi et al., 2013; Mulle et al., 2013; Turroni et al., 2014). Knowing that children with autism have digestion problems, deficiency in vitamin B12, and immune dysregulation and gut inflammation (Rossignol and Frye, 2012; Wasilewska and Klukowski, 2015; Zhang et al., 2016) it could be hypothesized that these problems could be associated with deficiency of intestinal *Bifidobacteria*.

Additionally, it was suggested that particular *Bifidobacterium* strains have the potential to treat some mental disorders, like depression, and anxiety. Treatment with *B. infantis* was shown to be beneficial in the treatment of depression, through elevation of the serotonergic precursor-tryptophan (Desbonnet et al., 2008), and that it can improve hypothalamic-pituitary axis mediated stress response (Sudo et al., 2004), while treatment with *B. longum* was shown to have an anxiolytic effect (Bercik et al., 2011).

It could be assumed that low *Bifidobacteria* diversity could be associated with antibiotic treatment in the first years of life, in line with results revealing the high incidence of opportunistic pathogens from *Clostridium* cluster. This assumption is in accordance with literature data describing the greater diversity of *Bifidobacterium* spp. in healthy children compared to antibiotic-treated infants (Hussey et al., 2011) and significantly higher oral antibiotic use in children with autism vs. typical children (Konstantareas and Homatidis, 1987; Niehus and Lord, 2006). Interestingly, our results revealed frequent occurrence of *Bifidobacterium animalis* subsp*. lactis* in all NDD groups, except in CHA, while it was not detected in any of the control samples, similar to the previously published data (Finegold et al., 2010). The higher frequency of *B. animalis* might be a consequence of frequent probiotic use in the patient group due to GI problems, since various strains of this species are commonly used as probiotics (Merenstein et al., 2015).

Three butyrate producing bacteria, *Faecalibacterium prausnitzii, Butyricicoccus pullicaecorum*, and *Eubacterium rectale*, were less frequent in the patients' groups. Butyric acid is the preferred source of energy for colonocytes and affects their proliferation, differentiation and apoptosis, has anti-inflammatory effects, reinforces the colonic defense barrier by increasing production of mucins and antimicrobial peptides and decreases intestinal epithelial permeability (Hamer et al., 2008; Van Immerseel et al., 2010). *Faecalibacterium prausnitzii* is one of the most abundant bacteria in the human gut ecosystem (Khan et al., 2012). *Faecalibacterium prausnitzii* and *Butyricicoccus pullicaecorum* are important suppliers of butyrate to the colonic epithelium and depletion of these bacteria was associated with irritable bowel syndrome (IBS) (Steppe et al., 2014), while it was shown that *B. pullicaecorum* strengthens epithelial barrier function (Eeckhaut et al., 2013). Another non-amylolytic member of the *Ruminococcacea* related to *F. prausnitzii, Ruminococcus champanellensis* (Ze et al., 2015), was less frequently found in the patients' groups, particularly in CHA and ERLD samples. *R. champanellensis* is the only human colonic bacterium reported to be capable of degrading crystalline cellulose and fermentation of dietary non-digestible carbohydrates. It has important consequences for health via modulation of microbiota composition by supplying energy and supporting microbial growth (Chassard et al., 2010; Ben David et al., 2015). A study of fecal microbiota of children with autism and PDD-NOS revealed that *Faecalibacterium, Ruminococcus*, and *Eubacteriaceae* were found to be present at the lowest level in fecal samples of children with autism (De Angelis et al., 2013), which is in accordance with our results.

Several molecular mechanisms that could explain the influence of microbiota on the brain and that could be involved in the pathogenesis of autism and other NDDs have been revealed so far. SCFAs, potent bioactive molecules, produced by bacterial fermentation of dietary carbohydrates within the gut, of which acetate, propionate, and butyrate are the most abundant (95%), can induce widespread effects on gut, brain, and behavior (Den Besten et al., 2013). In this study it was observed that a positive correlation between number of rDNA amplicons obtained with universal primers and level of propionic acid, as well as a trend for level of total SCFAs and butyric acid in the Control group, was lost in the patients' groups, leading to the conclusion that lower numbers of strong SCFA-producing bacteria are present in the microbiota of NDD patients.

It was found that physiological concentrations of SCFA promote epithelial barrier function in the large intestine (Suzuki et al., 2008). Acetate, propionate and butyrate alone or in combination stimulated the formation of tight junctions (TJ) (Feng et al., 2018) by increasing expression of TJ proteins (Hamer et al., 2008) through their histone deacetylase (HDAC) inhibitor activity (Ohata et al., 2005). Therefore, lower levels of SCFAs might disturb epithelial barrier function in the gut. Since decreased intestinal epithelial barrier function was found in some children with autism (D'eufemia et al., 1996; Boukthir et al., 2010; De Magistris et al., 2010), absence of strong SCFA-producing bacteria and lower levels of SCFAs in the gut could be the cause of this defect.

Along with the intestinal epithelial barrier dysfunction, increased blood-brain barrier permeability was detected in ASD patients (Fiorentino et al., 2016). It was discovered that normal gut microbiota have beneficial effects on stability of the blood-brain barrier (BBB) (Braniste et al., 2014). Increased BBB permeability in germ-free mice was associated with decreased levels of TJ proteins in several brain regions. These changes were restored to normal levels by colonization of germ-free animals with intestinal microbiota from pathogen-free mice (conventionalization), colonization with a single butyrate producing bacteria or oral gavage with sodium butyrate. SCFAs seem to play an important role in maintaining BBB integrity, which has a central role in brain development and the preservation of CNS homeostasis (Silva et al., 2020).

Besides influencing the intestinal epithelial barrier and BBB, it has been shown that microbial products, propionic and butyric acid, have strong epigenetic potential and can induce alterations in expression of genes involved in neurotransmitter systems, neuronal cell adhesion molecules, inflammation, oxidative stress, lipid metabolism and mitochondrial function, all of which have been associated with ASD development (Nankova et al., 2014). In addition, treatment with sodium-butyrate induced an increase in neurotrophin levels (BDNF, NGF, and GDNF) in the hippocampus and frontal cortex, which was associated with improvement in recognition memory (Valvassori et al., 2014), and reduction in hyperlocomotion (Varela et al., 2015) in rats. Sodium-butyrate treatments have also enhanced neurite outgrowth (Suzuki-Mizushima et al., 2002) and shown neuroprotective capacity (Wu et al., 2008). These findings imply that butyrate up-regulates expression of pro-survival, pro-regenerative and pro-plasticity genes by increasing histone acetylation around promoters of these genes (Bourassa et al., 2016).

Although in this study significant differences in the levels of SCFAs weren't detected, it was observed that the values for total SCFAs, as well as butyric acid, were lowest in the CHA group. Literature data revealed that total SCFA levels were significantly lower in autism disorder (AD) and PDD-NOS patients (Adams et al., 2011; De Angelis et al., 2013). Our assumption is that a significant difference in SCFA levels between the CHA subgroup and Control group in our study was not detected due to the small number of CHA patients (ratio CHA:NDD patients is 1: 7.2). This is to some extent in accordance with an estimation by the National Survey of Children's Health in the US for 2007, according to which 1.1% of children ages 3–17 years were reported to have ASDs, while 15% were affected by NDD (ratio 1:15). This estimation included other developmental delays, such as intellectual disability, cerebral palsy, seizures, stuttering or stammering, moderate to profound hearing loss, and blindness (Kogan et al., 2009; Boyle et al., 2011) that weren't included in this study.

As stated by other authors, a positive correlation was found between the level of total SCFAs and the levels of *Faecalibacterium, Ruminococcus*, and *Bifidobacterium* (De Angelis et al., 2013), which is in accordance with our observation that *F. prausnitzii* and *R. champanellensis* are present with lower frequency in the patient group, as were several strains from the *Bifidobacterium* genus. Although bacteria belonging to *Ruminococcus* and *Bifidobacterium* genera do not produce butyrate, they can promote the production of SCFAs thanks to their ability to process non-digestible carbohydrates and can stimulate butyrate production by cross-feeding of colonic butyrate-producing bacteria (Belenguer et al., 2006).

According to current knowledge, it can be assumed that SCFAs are important molecular mediators in the gut-brain axis and whose effects might be involved in development of autism-like symptoms (Rogers et al., 2016). Therefore, lower numbers of strong SCFA-producing bacteria in the gut microbiota of NDD patients, especially a lower incidence of butyrate producing bacteria, might have a significant impact on the etiology of these disorders.

Recently, it has been recognized that microbiota have a significant role in neurodevelopment due to the intense and dynamic bidirectional communication in the gut-brain axis, and that early-life perturbations of initial microbiota colonization can lead to mental disorders later in life (Diaz Heijtz et al., 2011; Borre et al., 2014; De Theije et al., 2014). Development of the microbiota occurs in parallel with neurodevelopment and they have similar critical developmental windows sensitive to disruption (O'mahony et al., 2017). It has been suggested that colonization by gut microbiota impacts mammalian brain development and subsequent adult behavior in part by altered expression of several genes (Diaz Heijtz et al., 2011). It has also been emphasized that a critical period might exist after which reconstitution of microbiota does not normalize the behavioral phenotype (Sudo et al., 2004; Diaz Heijtz et al., 2011; Neufeld K. A. et al., 2011; Desbonnet et al., 2014) or neurochemical disturbances that have occurred in early phases of brain development (Clarke et al., 2013).

## Conclusion

Our study reveals that the intestinal microbiota from NDD patients differs from the microbiota of healthy children. Lower bacterial diversity of beneficial commensal bacteria and increased incidence of resident microbes with pathogenic potential were detected. The alterations reported in the present study are partly consistent with other studies performed on children with autism and support the hypothesis that microbiota dysbiosis could be associated with GI disorders frequently observed in NDD patients and appearance of autism-like behavior (Finegold et al., 2002, 2010; Song Y. et al., 2004; Parracho et al., 2005; Adams et al., 2011; De Angelis et al., 2013; Tomova et al., 2015). The characterization of gut microbiota in our study, as in aforementioned studies, was performed according to analyses of fecal samples. Some authors have pointed out that there are differences in composition of fecal and mucosa associated microbiota in healthy individuals (Carstens et al., 2018), such that mucosal-associated microbiota better discriminate patients from controls (Altomare et al., 2019) and therefore deserve more attention (Ouwehand et al., 2004). Although this must be kept in mind, fecal samples are often used as representative of gut microbiota whenever colonic biopsies are not available.

Our results provide a rationale for further studies of gut microbiota dysbiosis in NDD patients and elucidation of possible pathogenic modes of action of specific bacteria. According to these results and literature data, we suggest that early life microbiome screening could be a potentially useful tool for prevention and treatment of GI disturbances and accompanied behavioral problems frequently observed in autism and other patients from a broad spectrum of NDD. In addition, correction of microbiota composition and supplementation with several health promoting strains, found to be deficient in these patients, could be a safe adjuvant therapy in the treatment of NDD accompanied by GI disturbances.

## Data Availability Statement

The raw data supporting the conclusions of this article will be made available by the authors, without undue reservation, to any qualified researcher.

## Ethics Statement

The study involving human participants was reviewed and approved by Ethics and Research Committee of Institute of Molecular Genetics and Genetic Engineering, University of Belgrade (O-EO-002/2015). Written informed consent to participate in this study was provided by the participants' parent.

## Author Contributions

KB contributed to taking interviews with parents, collecting anamnesis' data and the samples from patients, carrying out the interpretation of data, and preparing the first draft of the manuscript. ÐI contributed to making conception and design of the study, collecting samples from healthy children, carrying out the experiments, analysis and interpretation of data, preparing the first draft of the manuscript, performing checking, and editing of all revisions of the manuscript. SS contributed to carrying out the experiments, preparing the first draft of the manuscript. DV contributed to analysis and interpretation of the data, performing checking and editing of all revisions of the manuscript. MTom contributed to editing final revision of the manuscript. NG contributed to making a conception and design of the study, doing analysis and interpretation of data, performing checking, and editing of all revisions of the manuscript. MTol contributed to making conception, design and supervision of the study, carrying out the experiments, analysis and interpretation of data, preparing the first draft of the manuscript, performing checking, and editing of all revisions of the manuscript.

### Conflict of Interest

The authors declare that the research was conducted in the absence of any commercial or financial relationships that could be construed as a potential conflict of interest.
