# Supplementary material for: Gut Microbiota Dysbiosis Associated With Altered Production of Short Chain Fatty Acids in Children With Neurodevelopmental Disorders
Source: Front Cell Infect Microbiol. 2020 May 19;10:223. doi: 10.3389/fcimb.2020.00223 (PMC7248180; doi:10.3389/fcimb.2020.00223)
Supplement: Supplementary file 1 [file Table_1.docx]

**Supplementary Table 1.** **Clinical presentation of patients.**

| **Patient no** | **Clinical Picture** | **Dg** | **antibiotic**  **treatment** | **microbiota** | **GI disorders** |
| --- | --- | --- | --- | --- | --- |
| **1** | Not aware of actual environment, stereotypes | MSDD | + | *R. ilealis*, *I. bartlettii*,  *E. ramosum* |  |
| **2** | Withdrawn in his own world, sensory auto-stimulation, immature *g*raphomotor  or handwriting skills | MSDD |  | *R. ilealis,*  *I. bartlettii* | Stomach cramps |
| **3** | Newborn jaundice, frequent ear infections, short attention, stereotyped play, immature motor skills (gross and graphomotor), deficits in daily living skills | MSDD | + | *R. ilealis,*  *I. bartlettii*,  *E. ramosum* |  |
| **4** | Frequent ear infections, panic attacks, repetitive behavior, bizarre behavior, peripheral vision, unstable emotionally (crying and laughing without reason) sensory dysfunction, short attention, impulsivity | MSDD | + | *R. ilealis,*  *I. bartlettii*,  *E. ramosum* | Mushy stool several times a day |
| **5** | Macrocephaly, newborn jaundice, hypotonic | MSDD |  | *R. ilealis,*  *I. bartlettii*,  *E. ramosum* |  |
| **6** | Febrile convulsion, newborn jaundice, no breast-feeding, unstable emotionally (crying and laughing without reason), stereotype movement, low tolerance for frustration, sensory auto-stimulation, immature motor skills | MSDD |  | *R. ilealis,*  *I. bartlettii* |  |
| **7** | Hypotonic, low tolerance for frustration, anxiety | MSDD |  | *I. bartlettii*, *E. ramosum,*  no Bifidobacteria | periodical constipations |
| **8** | Impulsivity, lack of response to his name, hyperactivity, short attention, stereotypes | MSDD | + | *R. ilealis,*  *I. bartlettii* |  |
| **9** | Withdrawn in his own world, selective attention, sleeping disturbance, phobias | MSDD |  | *R. ilealis, I. bartlettii* |  |
| **10** | Reduced eye contact, attention deficit, withdrawn in his own world, sensory auto-stimulation, teeth grinding (bruxism) | MSDD |  | *R. ilealis,*  *I. bartlettii* | Gases |
| **11** | Newborn jaundice, not aware of actual environment, lack of response to his name, short attention, sleeping disturbance, reduced eye contact, stereotypes | MSDD |  | *R. ilealis,*  *I. bartlettii* |  |
| **12** | Attention disorder, stereotypes | MSDD |  | *D. guttoideum*, *R. ilealis* |  |
| **13** | Impulsive reactions, short attention, lack of response to his name , eczema, capricious, disorganized | PDD-NOS |  | *R. ilealis*,  *I. bartlettii* |  |
| **14** | Vocal stereotypes, flapping, increased pain threshold, peripheral vision, reduced eye contact, walks tiptoe, teeth grinding (bruxism) | PDD-NOS | + | *D*. *guttoideum*,  *R. ilealis*,  *I. bartlettii*,  *E. ramosum* | Opstipation |
| **15** | Hyperactivity, short attention  lack of response to his name | PDD-NOS |  | *R. ilealis*,  *I. bartlettii* | Mushy stool several times a day |
| **16** | Newborn jaundice, not breast-feeding, attention disorder (low vigility), deficit of pragmatic communication skills, immature  *g*raphomotor or handwriting skills, echolalia | PDD-NOS | + | *R. ilealis*, | Strong stomach cramps, diarrhea, mushy stool several times a day |
| **17** | Hyperactivity, short attention, selective attention, low tolerance for frustration,  impulsivity | PDD-NOS |  | *D*. *guttoideum*,  *R. ilealis*,  *I. bartlettii* |  |
| **18** | Low tolerance for frustration, short attention, impulsivity | PDD-NOS |  |  | Periodically mushy stool |
| **19** | Sensory dysfunction, low tolerance for frustration with impulsive and aggressive reactions, good motor skills, lack of response to his name, reduced eye contact | PDD-NOS |  | *R. ilealis*,  *I. bartlettii* |  |
| **20** | Hyperactivity, anxiety, low tolerance for frustration, sleeping disturbance, short attention | PDD-NOS |  | *D*. *guttoideum*, *R. ilealis,*  no Bifidobacteria | Stomach cramps |
| **21** | Short attention | PDD-NOS |  | *I. bartlettii,*  no Bifidobacteria |  |
| **22** | Newborn jaundice, withdrawn in his own world, selective attention, sensory auto-stimulation | PDD-NOS | + | *R. ilealis*,  *I. bartlettii* | Hard stool, painful to pass, delaying going to the toilet |
| **23** | Newborn jaundice, vocal stereotypes, reduced eye contact, repetitive body movements - flapping, hyperactivity, short attention, impulsivity | ERLD |  | *D*. *guttoideum*,  *R. ilealis*,  *I bartlettii*,  *E ramosum* | Several stools per day |
| **24** | Eczema, selective attention, low tolerance for frustration, lack of response to his name | ERLD |  | *D*. *guttoideum*,  *R. ilealis*,  *I. bartelttii* |  |
| **25** | Frequent ear infections, anxiety, low tolerance for frustration | ERLD |  | *R. ilealis, I. bartelttii,*  *E. ramosum* |  |
| **26** | Newborn jaundice, eczema, stereotyped play | ERLD |  | *R. ilealis, I. bartelttii,*  no Bifidobacteria | Opstipation |
| **27** | Newborn jaundice, eczema | ERLD |  | *D*. *guttoideum*, *R. ilealis,*  *I. bartelttii, E. ramosum* |  |
| **28** | Low tolerance for frustration,  sleeping disturbance,  eczema, irritability | ERLD |  | *D*. *guttoideum*, *R. ilealis,*  *I. bartelttii, E. Ramosum,*  no Bifidobacteria |  |
| **29** | Newborn jaundice, frequent ear infections, eczema, attention disorder, low tolerance for frustration with impulsive and aggressive reactions | ERLD | + | *I. barlettii* | A soft stool, unformed, with food remains |
| **30** | Newborn jaundice, impulsivity, attention disorder, sleeping disturbance, lack of response to his name, reduced eye contact | ERLD |  | *D*. *guttoideum*,  *R. ilealis,*  *I. bartelttii,*  *E. ramosum* |  |
| **31** | Frequent ear infections, impulsivity, low tolerance for frustration | ERLD | + | *R. ilealis,*  *I. bartlettii* | Normal stool every second day |
| **32** | Irritable, impulsive reactions, low tolerance for frustration, sensory dysfunction, newborn jaundice | CHA | + | *R. ilealis,*  *I. bartlettii,*  *E. ramosum* |  |
| **33** | Diabetes, not aware of actual environment, unstable emotionally (crying and laughing without reason), stereotypes, sleeping disturbances, low tolerance for frustration with impulsive and aggressive reactions | CHA |  | *D*. *guttoideum*, *I. bartlettii,*  no Bifidobacteria | Stomach cramps, abdominal pains, loose stool |
| **34** | Newborn jaundice, middle ear infections, lack of response to his name, increased pain threshold, low tolerance for frustration, withdrawn in his own world | CHA | + | *I. bartlettii,*  *R. ilealis* |  |
| **35** | Lack of response to his name, sleeping disturbance, teeth grinding (bruxism), short attention, hyperactivity | CHA |  | *D*. *guttoideum*,  *I. bartlettii* |  |
| **36** | Low tolerance for frustration, impulsivity, short attention, unstable emotionally (crying and laughing without reason) | CHA | + | *R. ilealis*,  *I bartlettii,*  *E. ramosum* | Mushy stool |

Clinical characteristics of NDD patients, possibly linked with dysbiosis are presented. Perinatal factors that could have influence on microbiota composition (caesarian section, newborn jaundice, no breast-feeding) so as presence of frequent infections were noted. Frequent application of antibiotic therapy was detected in 12/36 patients according to the interview with parents, whilst no antibiotic intake for at least three months prior to entering the study was one of the main inclusion criteria. Presence of particular gastro-intestinal disturbances was specified. Results of microbiota analyses from this study were displayed - presence of *Clostridium*-like bacteria or complete deficiency of beneficial bacteria (*Bifidobacteria*).
