# Supplementary material for: Gut Microbiota Dysbiosis Associated With Altered Production of Short Chain Fatty Acids in Children With Neurodevelopmental Disorders
Source: Front Cell Infect Microbiol. 2020 May 19;10:223. doi: 10.3389/fcimb.2020.00223 (PMC7248180; doi:10.3389/fcimb.2020.00223)
Supplement: Supplementary file 2 [file Table_2.docx]

**Supplementary Table 2**: **HPLC – UV gradient elution program for the analysis of SCFAs in human fecal extracts**

| Time (min) | MF A (%) | MF B (%) | Flow rate (ml/min) |
| --- | --- | --- | --- |
| 0 | 100 | 0 | 0,8 |
| 3.5 | 100 | 0 | 0.8 |
| 4 | 92.5 | 7.5 | 0.4 |
| 7.5 | 92.5 | 7.5 | 0.4 |
| 8 | 85 | 15 | 0.4 |
| 10.5 | 85 | 15 | 0.4 |
| 11 | 80 | 20 | 0.4 |
| 13 | 80 | 20 | 0.4 |
| 13.5 | 80 | 20 | 0.4 |
| 20 | 75 | 25 | 0.4 |
| 20.5 | 100 | 0 | 1.2 |
| 23.5 | 100 | 0 | 1.2 |
| 24 | 100 | 0 | 0.8 |
| 25 | 100 | 0 | 0.8 |

Mobile phase (MF) A: 20 mM NaH2PO4 adjusted to pH 2.2 using phosphoric acid; mobile phase B: acetonitrile. Chromatographic separation was tested on Hypersil Gold aQ column (150 mm × 4.6 mm i.d.) with particle size of 3 µm.
