# Supplementary material for: Gut Microbiota Dysbiosis Associated With Altered Production of Short Chain Fatty Acids in Children With Neurodevelopmental Disorders
Source: Front Cell Infect Microbiol. 2020 May 19;10:223. doi: 10.3389/fcimb.2020.00223 (PMC7248180; doi:10.3389/fcimb.2020.00223)
Supplement: Supplementary file 3 [file Table_3.docx]

**Supplementary Table 3. The values of Dice analysis**

|  | **Mean** | **St. dev** |
| --- | --- | --- |
| **Uni primers** |  |  |
| MSDD | 63.55 | 17.67 |
| PDD-NOS | 67.56 | 14.94 |
| ERLD | 66.54 | 12.63 |
| CHA | 61.78 | 10.43 |
| NDD | 65.27 | 14.62 |
| CTRL | 65.82 | 15.46 |
| **LB-specific primers** |  |  |
| MSDD | 51.77 | 18.74 |
| PDD-NOS | 43.60 | 19.35 |
| ERLD | 31.12 | 22.30 |
| CHA | 50.17 | 23.56 |
| NDD | 47.24 | 19.60 |
| CTRL | 59.30 | 15.21 |
| **BB-specific primers** |  |  |
| MSDD | 49.88 | 28.56 |
| PDD-NOS | 36.85 | 39.15 |
| ERLD | 30.13 | 37.50 |
| CHA | 52.00 | 45.41 |
| NDD | 41.75 | 35.77 |
| CTRL | 71.88 | 16.16 |
| **All three sets of primers** |  |  |
| MSDD | 57.80 | 12.52 |
| PDD-NOS | 55.65 | 12.47 |
| ERLD | 49.90 | 13.64 |
| CHA | 58.53 | 12.19 |
| NDD | 55.42 | 13.02 |
| CTRL | 64.57 | 9.353 |
| **Uni primers** |  |  |
| MSDD/PDD-NOS | 63.46 | 17.88 |
| MSDD/ERLD | 64.90 | 16.64 |
| MSDD/CHA | 64.71 | 15.15 |
| PDD-NOS/ERLD | 62.91 | 12.34 |
| PDD-NOS/CHA | 64.72 | 13.52 |
| ERLD/CHA | 66.55 | 10.02 |
| **LB-specific primers** |  |  |
| MSDD/PDD-NOS | 49.49 | 20.50 |
| MSDD/ERLD | 38.95 | 22.07 |
| MSDD/CHA | 51.55 | 19.89 |
| PDD-NOS/ERLD | 38.25 | 20.43 |
| PDD-NOS/CHA | 48.94 | 20.94 |
| ERLD/CHA | 36.37 | 21.96 |
| **BB-specific primers** |  |  |
| MSDD/PDD-NOS | 43.00 | 35.25 |
| MSDD/ERLD | 41.01 | 35.68 |
| MSDD/CHA | 52.85 | 35.89 |
| PDD-NOS/ERLD | 36.08 | 38.81 |
| PDD-NOS/CHA | 42.67 | 41.18 |
| ERLD/CHA | 41.14 | 40.90 |
| **All three sets of primers** |  |  |
| MSDD/PDD-NOS | 56.84 | 14.17 |
| MSDD/ERLD | 52.74 | 14.50 |
| MSDD/CHA | 59.58 | 12.16 |
| PDD-NOS/ERLD | 51.90 | 11.20 |
| PDD-NOS/CHA | 58.29 | 11.55 |
| ERLD/CHA | 54.15 | 10.58 |
| **Uni primers** |  |  |
| MSDD/CTRL | 60.62 | 17.46 |
| PDD-NOS/CTRL | 64.89 | 13.03 |
| ERLD/CTRL | 59.29 | 15.76 |
| CHA/CTRL | 62.15 | 12.70 |
| NDD/CTRL | 61.84 | 15.43 |
| **LB-specific primers** |  |  |
| MSDD/CTRL | 51.18 | 15.97 |
| PDD-NOS/CTRL | 50.32 | 16.94 |
| ERLD/CTRL | 40.64 | 20.99 |
| CHA/CTRL | 47.79 | 16.79 |
| NDD/CTRL | 47.83 | 18.21 |
| **BB-specific primers** |  |  |
| MSDD/CTRL | 55.90 | 24.67 |
| PDD-NOS/CTRL | 46.09 | 34.65 |
| ERLD/CTRL | 46.16 | 35.38 |
| CHA/CTRL | 57.35 | 32.66 |
| NDD/CTRL | 50.94 | 31.95 |
| **All three sets of primers** |  |  |
| MSDD/CTRL | 56.95 | 11.74 |
| PDD-NOS/CTRL | 57.00 | 8.827 |
| ERLD/CTRL | 50.88 | 14.32 |
| CHA/CTRL | 56.50 | 10.75 |
| NDD/CTRL | 55.38 | 10.59 |
