# Supplementary material for: Gut Microbiota Dysbiosis Associated With Altered Production of Short Chain Fatty Acids in Children With Neurodevelopmental Disorders
Source: Front Cell Infect Microbiol. 2020 May 19;10:223. doi: 10.3389/fcimb.2020.00223 (PMC7248180; doi:10.3389/fcimb.2020.00223)
Supplement: Supplementary file 4 [file Table_4.docx]

**Supplementary Table 4:** **Appearance of identified bacterial strains in NDD patient subgroups (different diagnoses) and Control group**.

| **Bacterial species obtained with**  **universal primers** | MSDD (%)  n=12 | PDD-NOS (%)  n=10 | ERLD (%)  n=9 | CHA (%)  n=5 | CTRL (%)  n=28 |
| --- | --- | --- | --- | --- | --- |
| *E. ramosum* strain JCM 1298 | 41.7 | 10,0 | 55.6 | 40,0 | 25,0 |
| *I. bartlettii* strain WAL 16138 | 100,0^*^ | 70,0 | 100,0 | 100,0 | 64.3 |
| *R. ilealis* strain CRIB (2) | 91.7^**^ | 80,0^*^ | 88.9^*^ | 60,0 | 32.1 |
| *D. guttoideum* strain DSM 402 | 16.7 | 30,0 | 55.6^**^ | 40,0 | 3.6 |
| **Bacterial species obtained with**  **LB-specific primers** |  |  |  |  |  |
| *E. faecalis* strain NBRC 100480 | 58.3 | 60,0 | 55.6 | 60,0 | 89.3 |
| *E. rectale* strain ATCC 33656 | 33.3 | 20,0 | 22.2 | / | 46.4 |
| *S. pasteurianus* | /^*^ | 20,0 | 33.3 | / | 42.9 |
| *B. pullicaecorum* strain 25-3 | /^*^ | 20,0 | 33.3 | / | 42.9 |
| *D. invisus* strain JCM 17566 | /^*^ | 20,0 | 33.3 | / | 42.9 |
| *R. champanellensis* strain 18P13 | 16.7 | 20,0 | 11.1 | / | 53.6 |
| *E. gallinarum* strain LMG 13129 | 16.7 | 20,0 | 11.1 | / | 53.6 |
| *L. sakei* subsp. *carnosus* strain CCUG 31331 | 41.7 | 30,0 | 22.2^*^ | 20,0 | 67.9 |
| *F. prausnitzii* strain ATCC 27768 | 41.7 | 30,0 | 22.2^*^ | 20,0 | 67.9 |
| *L. rhamnosus* strain NBRC 3425 | /^**^ | 20,0 | 11.1 | 20,0 | 42.9 |
| *L. sakei* (2) | 50,0 | 60,0 | 11.1^**^ | 80,0 | 75,0 |
| *D. invisus* strain JCM 17566 (2) | 41.7^*^ | 40,0^*^ | 33.3^*^ | 40,0 | 82.1 |
| **Bacterial species obtained with**  **BB-specific primers** |  |  |  |  |  |
| *B. pseudocatenulatum* strain B1279 | 16.7 | 10,0 | 22.2 | / | 35.7 |
| *B. adolescentis* strain ATCC 15703 | / | / | / | / | 28.6 |
| *B. animalis* subsp. *lactis* strain YIT 4121 | 16.7 | 40,0^**^ | 11.1 | / | / |
| *B. faecale* | 91.7 | 70,0^*^ | 66.7^*^ | 80,0 | 100 |
| *B. longum* subsp. *infantis* strain ATCC 15697 | 50,0 | 60,0 | 55.6 | 40,0 | 71.4 |
| *B. stercoris* strain Eg1 16S ribosomal | 66.7^**^ | 60,0^**^ | 66.7^*^ | 80,0 | 100,0 |

Comparisons between patient subgroups (different diagnoses) and Control group for each bacterial strain were performed with Pearson’s Chi-square test. n – number of participants per group. Results were considered statistically significant at P<0.05. ^*^P<0.05; ^**^P<0.01
